# Associations between inflammation, coagulation, cardiac strain and injury, and subclinical vascular disease with frailty in older men: a cross-sectional study

**DOI:** 10.1186/s12877-022-03106-3

**Published:** 2022-05-09

**Authors:** Douglas G. J. McKechnie, Meera Patel, A. Olia Papacosta, Lucy T. Lennon, Elizabeth A. Ellins, Julian P. J. Halcox, Sheena E. Ramsay, Peter H. Whincup, S. Goya Wannamethee

**Affiliations:** 1 Department of Primary Care and Population Health, University College London, London, NW3 2PF UK; 2 Institute of Life Science, Swansea University, Swansea, UK; 3 Population Health Sciences Institute, Newcastle University, Newcastle upon Tyne, UK; 4 Population Health Research Institute, St George’s University of London, London, UK

**Keywords:** Frailty, Biomarkers, Cardiovascular diseases, Aging, Atherosclerosis

## Abstract

**Background:**

Inflammation, coagulation activation, endothelial dysfunction and subclinical vascular disease are cross-sectionally associated with frailty. Cardiac-specific biomarkers are less-well characterised. We assessed associations between these and frailty, in men with, and without, cardiovascular disease (CVD).

**Methods:**

Cross-sectional analysis of 1096 men without, and 303 with, CVD, aged 71–92, from the British Regional Heart Study. Multinominal logistic regression was performed to examine the associations between frailty status (robust/pre-frail/frail) and, separately, C-reactive protein (CRP), interleukin-6 (IL-6), tissue plasminogen activator (tPA), D-dimer, von Willebrand factor (vWF), high-sensitivity cardiac troponin-T (hs-cTnT), N-terminal pro B-type natriuretic peptide (NT-proBNP) (all natural log-transformed), and, in men without CVD, carotid intima-media thickness (CIMT), carotid-femoral pulse wave velocity (cfPWV), carotid distensibility coefficient (DC), and ankle-brachial pressure index (ABPI), adjusted for age, renal function, BMI, social class, smoking, polypharmacy, cognition, multimorbidity and systolic blood pressure. Explanatory variables with *p* < 0.05 were carried forward into mutually-adjusted analysis.

**Results:**

In men without CVD, higher CRP, IL-6, vWF, tPA, hs-cTnT, NT-proBNP, cfPWV, and lower DC were significantly associated with frailty; mutually-adjusted, log IL-6 (OR for frailty = 2.02, 95%CI 1.38–2.95), log hs-cTnT (OR = 1.95, 95%CI 1.24–3.05) and DC (OR = 0.92, 95%CI 0.86–0.99) retained associations. In men with CVD, higher CRP, IL-6, and hs-cTnT, but not vWF, tPA, NT-proBNP or D-dimer, were significantly associated with frailty; mutually-adjusted, log hs-cTnT (OR 3.82, 95%CI 1.84–7.95) retained a significant association.

**Conclusions:**

In older men, biomarkers of myocardial injury are associated with frailty. Inflammation is associated with frailty in men without CVD. Carotid artery stiffness is associated with frailty in men without CVD, independently of these biomarkers.

## Background

Frailty describes a state of extreme vulnerability to pathophysiological insults [1]. It is associated with myriad adverse events, such as falls, dependency and need for care, hospitalisation and early mortality, [2] and its prevalence is likely to increase as populations age globally. Frailty is more common in older people with cardiovascular disease (CVD) [3], and both CVD and frailty show bidirectional longitudinal relationships: people with frailty are more likely to develop CVD [3], and people with CVD may be more likely to become frail [4, 5].

Understanding the shared biological basis of the two conditions could help understand why these commonly coexist, and how they interact with one another; these may eventually lead to targeted therapeutic interventions. Chronic inflammation [6, 7], coagulation system activation [8], endothelial dysfunction [9], and neurohormonal activation [10] have all been proposed as common pathophysiological pathways, as has atherosclerosis. Several population studies have shown associations between frailty status and markers of inflammation (C-reactive protein (CRP) and interleukin-6 (IL-6)), markers of coagulation activation (D-dimer, factor VIII [8] and fibrinogen [6]) and biomarkers of endothelial dysfunction, such as asymmetric dimethylarginine, and flow mediated dilation, an ultrasound parameter that reflects endothelial function [11, 12].

Associations between neurohormonal activation and frailty have been less well-characterised, although there are suggestions that B-type natriuretic peptide (BNP) levels are associated with frailty [13, 14]. High-sensitivity cardiac troponin T (hs-cTnT) is a sensitive blood marker of myocardial injury of any cause, including ischaemic and inflammatory myocardial insults and is associated with cardiovascular risk and death [15]. High sensitivity cardiac troponin I has been associated with frailty in older adults with diabetes [16]; less has been studied of cardiac troponins in the general population.

Atherosclerosis can be measured non-invasively in its early stages, prior to the development of clinically overt cardiovascular disease. Such measures include ankle-brachial pressure index (ABPI), carotid-femoral pulse wave velocity (cfPWV), and carotid intima media thickness (CIMT), all of which have been associated with frailty and aspects of the frailty phenotype in cross-sectional studies [17–22], though these associations have not been reported in all studies [23, 24].

We therefore aimed, in a cross-sectional study of older British men [25], to examine associations between frailty and prefrailty with markers of inflammation (CRP and IL-6); endothelial dysfunction (von Willebrand factor (vWF)); coagulation (D-dimer and tPA); and myocardial injury (hs-cTnT) and myocardial strain (NT-proBNP) separately in men with, and without, overt clinical cardiovascular disease, to assess patterns of association in the two groups. We also examined the association between subclinical vascular disease (cfPWV, carotid artery distensibility (DC), CIMT, and ABPI) and frailty in men without overt cardiovascular disease, and determined the relative strength of associations between biomarkers, imaging markers, and frailty, accounting for one another.

## Methods

The British Regional Heart Study is a prospective cohort study, initiated in 1978–1980 and consisting of a socio-economically representative population of 7735 British men aged 40–59 years. They were selected from one general practice in each of 24 geographically-representative British towns [25]. In 2010–2012, all surviving men (*n* = 3137), then aged 71–92 years, were asked to complete a postal questionnaire and invited to attend a re-examination. The questionnaire was completed by 2137 men (68%) and included questions on medical history and lifestyle behaviour. A total of 1722 (55%) men attended the examination at an allocated time between 0800 and 1800 hours. A venous blood sample was collected after a minimum 6 hour fast using the Sarstedt Monovette system and stored at − 70 °C.

### Cardiovascular risk factors

In this cohort, details on the classification and measurements of alcohol intake, smoking status and physical activity assessed by questionnaire have been previously described [26]. Social class was defined as manual or non-manual occupation, as described previously [27]. Anthropometric measurements of body weight and height were carried out to calculate the body mass index (BMI) as weight/(height)^2^ (kg/m^2^). As a U-shaped relationship between BMI and frailty has been previously reported, [28] participants were divided into four groups: < 20 kg/m^2^, 20–24.9 kg/m^2^, 25–29.9 kg/m^2^, and ≥ 30 kg/m^2^. Physical performance tests included a timed walking test, where the time taken (in seconds) to walk 3 m at the participant’s normal walking pace was recorded and an assessment of grip strength, measured three times for each hand in kilograms using a Jamar Hydraulic Hand Dynamometer. The highest of the six readings was used for analysis. Presence of CVD (stroke, heart failure or myocardial infarction) or peripheral vascular disease (PVD) was based on the participants reporting a medical practitioner’s diagnosis in the questionnaire or through annually obtained general practice records. The diagnosis of CVD was always validated against hospital records. Men who reported a doctor diagnosis of diabetes or those with a fasting blood glucose ≥7 mmol/l were considered to have diabetes. Estimated glomerular filtration rate (eGFR) was calculated from the Modification of Diet in Renal Disease equation [29].

### Cognitive testing

Cognitive skills were assessed using the Test Your Memory (TYM) instrument [30] and diagnoses of ‘mild’ or ‘severe’ cognitive impairment made.

The TYM is a 10-task self-administered test that assesses orientation, copying, semantic knowledge, calculation, verbal fluency, similarities, naming, visuospatial abilities, and recall of a copied sentence. Scores below 33 were defined as ‘severe cognitive impairment’, and scores between 33 and 45 (if older than 80 years) or 33 and 46 (if younger than 80 years of age) were defined as ‘mild cognitive impairment’.

### Multimorbidity

As part of the baseline questionnaire, participants were asked if they had ever been told by a doctor that they had any of the following: cancer (at any site); anaemia; asthma; bronchitis; cataracts; chronic kidney disease; Crohn’s disease; depression; emphysema; gall bladder disease; gastric, peptic, or duodenal ulcers; glaucoma; gout; liver disease, cirrhosis or hepatitis; macular degeneration; osteoporosis; Parkinson’s disease; pneumonia; “prostate trouble”; shingles; ulcerative colitis; arthritis; a deep vein thrombosis and/or pulmonary embolus; and claudication.

The total number of reported comorbidities, plus diabetes mellitus and severe cognitive impairment (as defined elsewhere in Methods) were summed, without weighting, for each participant. Myocardial infarction, stroke, and heart failure were not included in this total.

### Polypharmacy

Questionnaire respondents provided a list of all regular medications. Polypharmacy was defined as the use of five or more regular medications [31].

### Blood markers

Measurements of CRP (mg/L), IL-6 (pg/mL), vWF (IU/dL), D-dimer (ng/mL), tPA (ng/mL), NT-proBNP (pg/mL) and hs-cTnT (pg/mL) were taken using fasting venous blood samples. An ultrasensitive assay was used to assess CRP (coefficient of variation; CV 6.9%), NT-proBNP and hs-cTnT on an automated clinically validated analyser (e411, Roche, Burgess Hill, UK) using the manufacturer’s calibrators’ and quality control reagents. For NT-proBNP and hs-cTnT, the lower limit of sensitivity was 5 pg/ml and 3 pg/ml respectively. The low and high controls CV of these cardiac markers were 6.7 and 4.9%. Enzyme-linked immunosorbent assays were used to measure the plasma levels of high sensitivity IL-6 (R&D systems, Oxon, UK), vWF (Technozym assay, Pathway Diagnostics, Dorking, UK), D-dimer and tPA (Asserachrom assays, Stago, Theale, UK). Intra and inter-assay CV of these markers were; 5.9 and 11.6% (IL-6), 14.1 and 14.3% (vWF), 5.4 and 3.2% (D-dimer), 5.5 and 4.1% (tPA).

### Non-invasive vascular markers

Two technicians measured the non-invasive vascular markers in series. Images of the left and right carotid arteries were obtained with a 5–10 mHz linear probe using a Z.one Ultra ultrasound system (Zonare Medical Systems, Mountain View, CA). Longitudinal images of the common carotid artery approximately 1 cm proximal to the carotid bifurcation and a cross-sectional sweep from the base of the common carotid artery to the jaw bone were recorded. Using the Carotid Analyser software (Medical Imaging Applications, Iowa City, IA), CIMT (distance between the leading edge of the intima and the media-adventitia interface) and the peak systolic and end-diastolic common carotid artery diameter were measured. A 5–10 mm plaque free area of interest, at least 1 cm from the bifurcation was selected from the longitudinal images. A mean CIMT was calculated from individual measurements obtained from the three end-diastolic images on each side. Mean distension was calculated from the maximum and minimum carotid artery diameter assessed from three consecutive waveforms. Using these measurements, the distensibility coefficient (DC) was measured with the following formula: DC = [(2x mean distension/baseline diameter)/mean pulse pressure (kPa)]*1000 [32]. With regard to the inter- and intra-observer reproducibility, the CV was 7.1 and 5.1% for CIMT (*n* = 109) and 9.2 and 11.9% for DC (*n* = 109).

Carotid to femoral pulse wave velocity (cfPWV) was measured using a Vicorder device (Skidmore Medical, UK). An inflatable bladder attached to a neck collar was positioned over the right carotid pulse, and a Hokanson SC10 cuff was placed around the middle of the right thigh. cfPWV length was measured from the sternal notch to the centre of the thigh cuff. The cuffs were then simultaneously inflated. The pressure waveforms were visually assessed so that a minimum of 3 good quality waveforms were taken. Two recordings were taken with a difference in cfPWV ≤0.5 m/s and averaged.

Ankle-brachial pressure indices (ABPIs) were measured using a Vicorder device (Skidmore Medical, UK), in the right and left sides sequentially. Hokanson SC10 cuffs were positioned on the upper arm and lower leg (above the ankle). Photoplethysmography sensors were then clipped to the end of the middle finger and the great toe. Brachial and tibial arteries were occluded simultaneously, as the cuffs were inflated to 180 mmHg. As the cuffs slowly deflated, the pulse data was visually assessed to minimise artefact from movement and to ensure that the blood pressures were taken at the point of the pulse returning at both sites. The Vicorder device provided blood pressures for both the brachial and ankle, and the ABPI ratio. Optimally, two measurements were recorded with a difference of ≤5 mmHg in either the brachial or the ankle pressures, and the mean value used. If this could not be achieved, three measures were taken and averaged.

### Frailty assessment

Using the data from both the questionnaire and examination, frailty and pre-frailty were defined according to the following components of the Fried frailty phenotype: (1) unintentional weight loss defined as ≥5% decrease over 4 years in self-reported weight that was stated as unintentional; (2) weakness defined as being in the lowest quintile of grip strength distribution; (3) low physical activity was assessed using self-reported questions on being less or much less active than an average man; (4) exhaustion was defined as answering “no” to “Do you feel full of energy?”; and (5) slow walking speed was defined as being in the lowest quintile of walking speed distribution or, if this was unavailable, self-report - any of: a) self-report of slow walking pace plus an inability to walk more than < 200 yards or difficulty walking across the room, b) self-report of slow walking pace plus at least some difficulty walking across a room, or c) self-report of being able to walk only a few steps unaided. Amongst all men who attended the examination and had both measured and self-reported walk speed data, 337 men fell into the lowest quartile of measured walk speed, and 123 of those met the self-report criteria for low walking speed. Men with three or more of these components were defined as frail, those with 1–2 components as pre-frail, and those with none, as robust.

### Exclusion criteria

Men missing data on any of: frailty; body mass index; cognition; smoking status; systolic blood pressure; eGFR; and/or polypharmacy were excluded from analysis, as were men who were missing all of the explanatory variables of interest (blood biomarkers and imaging parameters for men without CVD, and blood biomarkers for men with CVD). Missing data were handled using complete-case analysis, i.e. participants missing any variable used in each statistical analysis were excluded.

### Statistical analyses

We hypothesised that, if any causative link did indeed exist between the variables of interest, the direction of effect was more likely to be towards producing frailty (i.e. that inflammation, cardiac dysfunction, subclinical vascular disease, etc. might cause frailty, rather than vice versa). Frailty was therefore modelled as the dependent variable with the others modelled as explanatory variables.

Natural log transformation of CRP, IL-6, vWF, D-dimer, tPA, NT-proBNP and hs-cTnT were used as their distributions were highly skewed. Frailty status distributions were compared between men with, and men without CVD using the chi-square test. The two groups (with/without CVD) were subsequently analysed separately. To analyse the trend across the spectrum of frailty features, comparisons were made with frailty status as an ordinal variable – robust/pre-frail/frail - using one-way ANOVA for continuous variables and the Kruskall-Wallis test for categorical variables.

Stepwise multinomial logistic regression was performed, with frailty status (robust/pre-frail/frail) as the dependent variable. In the first set of analyses, log CRP, log IL-6, log vWF, log d-dimer, log tPA, log NT-proBNP, log hs-cTnT, DC, cfPWV, CIMT (all continuous variables), ABPI < 0.9 (yes/no) and ABPI > 1.4 (yes/no) were separately analysed as independent variables. All analyses were adjusted also for potential confounders:: age, systolic blood pressure, eGFR, number of non-cardiovascular comorbidities (all continuous), social class (manual/non-manual occupation), polypharmacy (yes/no), cognitive impairment (none/mild/severe), smoking status (never/recent ex-smoker/long-term ex-smoker/current), diabetes mellitus at baseline (yes/no), BMI group (< 20 kg/m^2^/20–24.9 kg/m^2^/25–29.9 kg/m^2^/≥30 kg/m^2^).

To examine the relative associations of the variables of interest with frailty, those variables that were statistically significantly associated with frailty status (at *p* < 0.05) were carried forward into a mutually-adjusted analysis, adjusted for each other and age/social class/smoking status/polypharmacy/comorbidity number/cognition/diabetes mellitus/BMI group and eGFR.

In the group of men with prevalent CVD, these analyses were repeated in the same way, with the exception of the subclinical atherosclerosis markers (DC, cfPWV, CIMT, ABPI), which were not included. These are markers of early, asymptomatic CVD, and therefore of limited use within this analysis in people with clinically-apparent CVD.

All analyses were performed using SAS version 9.3 (SAS, Cary, North Carolina).

## Results

Of the 3137 men from the original cohort who were alive and UK-resident at the time of the baseline examination, 1722 men (55%) attended. Complete data on relevant exposures (as adjusted for in the first stage of the multivariate analyses) were available for 1399 (81%) of attendees, who were used as the basis for all following analyses. 303 of these had clinically-apparent CVD. 82/303 (27%) and 168/303 (55%) of men with CVD were frail and pre-frail respectively, compared to 152/1096 (14%) and 603/1096 (55%) of men without CVD; distributions of frailty status in the CVD and non-CVD groups were highly statistically significantly different (*p* < 0.0001).

### Baseline characteristics

Baseline characteristics for men with and without prevalent CVD are summarized in Table 1. In both groups, men with pre-frailty, and, more so, frailty, tended to be older, have a higher prevalence of underweight and overweight men, polypharmacy and peripheral vascular disease. A higher proportion of frail men had diabetes in both groups, but the distribution of diabetes amongst frailty groups were statistically significantly different only in the CVD-free group. Frail men with CVD tended to have lower diastolic and systolic blood pressures compared to robust men.

**Table 1:** Baseline characteristics of individuals with, and without, clinically-apparent cardiovascular disease, by frailty status

	Free of clinically-apparent cardiovascular disease (***n*** = 1096)				Has clinically-apparent cardiovascular disease (***n*** = 303)			
	Robust (***n*** = 341)	Pre-frail (***n*** = 603)	Frail (***n*** = 152)	*p* for trend*	Robust (***n*** = 53)	Pre-frail (***n*** = 168)	Frail (***n*** = 82)	*p* for trend*
**Age (years)**	77 (3.6)	78 (4.5)	81 (5.3)	< 0.0001	78 (4.4)	78 (4.1)	80 (5.4)	0.003
**Manual occupation**	146 (43%)	263 (44%)	73 (48%)	0.37	25 (47%)	74 (44%)	46 (56%)	0.20
Never smoked	153 (45%)	239 (40%)	47 (31%)	0.04	16 (30%)	57 (34%)	21 (26%)	0.50
Long-term ex-smoker	164 (48%)	316 (52%)	92 (61%)		34 (64%)	98 (58%)	56 (68%)	
Recent ex-smoker	13 (4%)	26 (4%)	9 (6%)		3 (6%)	6 (4%)	2 (2%)	
Current smoker	11 (3%)	22 (4%)	4 (3%)		0	7 (4%)	3 (4%)	
**Physical measurements**								
Systolic blood pressure (mmHg)	148 (18)	147 (18)	143 (20)	0.07	145 (19)	141 (20)	136 (23)	0.01
Diastolic blood pressure (mmHg)	78 (11)	77 (11)	75 (12)	0.10	75 (11)	73 (11)	69 (12)	0.001
BMI < 20 kg/m^2^	3 (0.9%)	13 (2%)	6 (4%)	0.007	0	3 (2%)	6 (7%)	0.01
BMI 20–24.9 kg/m^2^	118 (35%)	162 (27%)	41 (27%)		17 (32%)	36 (21%)	16 (20%)	
BMI 25–29.9 kg/m^2^	174 (51%)	311 (52%)	76 (50%)		28 (53%)	92 *55%)	38 (46%)	
BMI ≥ 30 kg/m^2^	46 (13%)	117 (19%)	29 (19%)		9 (15%)	37 (22%)	22 (27%)	
**Comorbidities**								
Mild cognitive impairment	119 (35%)	246 (41%)	75 (49%)	0.005	20 (38%)	69 (41%)	36 (44%)	0.14
Severe cognitive impairment	25 (7%)	44 (7%)	14 (9%)		5 (9%)	17 (10%)	14 (17%)	
Diabetes mellitus	42 (12%)	83 (14%)	36 (24%)	0.007	10 (19%)	34 (20%)	21 (26%)	0.30
eGFR	77 (14)	74 (18)	69 (20)	< 0.0001	73 (16)	68 (20)	65 (19)	0.05
Peripheral vascular disease	2 (0.6%)	24 (4%)	7 (5%)	0.006	2 (4%)	8 (5%)	10 (12%)	0.03
Prior myocardial infarction	–	–	–		37 (70%)	114 (68%)	50 (61%)	0.24
Heart failure	–	–	–		9 (17%)	37 (22%)	34 (41%)	0.0005
Prior stroke	–	–	–		14 (26%)	49 (29%)	27 (33%)	0.40
**Total number of comorbidities (not including MI, HF or stroke)**								
Zero or one	140 (41%)	198 (33%)	23 (15%)	< 0.0001	20 (38%)	35 (21%)	8 (10%)	< 0.0001
Two, three or four	174 (51%)	321 (56%)	80 (53%)		31 (58%)	98 (58%)	45 (55%)	
Five or more	27 (8%)	84 (14%)	49 (32%)		2 (4%)	35 (21%)	29 (35%)	
**Taking five or more regular medications**	71 (21%)	191 (32%)	73 (48%)	< 0.0001	29 (55%)	112 (67%)	61 (74%)	0.02
**Inflammatory biomarkers**								
CRP (mg/L)	1.0 (0.5–2.0)	1.4 (0.7–3.1)	1.9 (0.7–5.1)	< 0.0001†	0.8 (0.3–1.9)	1.5 (0.7–3.0)	2.0 (0.7–5.2)	0.0002†
IL-6 (pg/mL)	2.3 (1.5–3.4)	3.1 (1.9–4.6)	3.9 (2.2–6.2)	< 0.0001†	2.7 (1.6–4.1)	3.2 (1.8–4.7)	4.6 (2.5–7.2)	< 0.0001†
**Coagulation biomarkers**								
tPA (ng/mL)	8.3 (6.1–11)	9.1 (6.7–12)	10.0 (7.5–13)	< 0.0001†	8.6 (6.6–10.8)	9.5 (7.0–12.5)	9.1 (6.4–12.8)	0.36†
D-dimer (ng/mL)	198 (140–270)	233 (158–314)	263 (165–412)	< 0.0001†	248 (143–337)	239 (144–326)	306 (218–434)	0.03†
**Endothelial biomarkers**								
vWF (IU/dL)	107 (79–133)	119 (91–143)	131 (101–177)	< 0.0001†	120 (88–142)	127 (91–177)	144 (100–227)	0.11†
**Cardiac biomarkers**								
hs-cTnT (pg/mL)	8.4 (6.1–13)	10.5 (7.2–15)	15.0 (9.1–23)	< 0.0001†	10 (6.5–16.4)	11 (7.2–16.4)	20 (12.8–32.5)	< 0.0001†
NT-proBNP (pg/mL)	87 (51–156)	123 (64–244)	198 (97–492)	< 0.0001†	227 (115–516)	196 (89–572)	479 (162–1700)	0.0001†
**Imaging**								
CIMT (mm)	0.79 (0.2)	0.80 (0.2)	0.83 (0.2)	0.001	–	–	–	–
cfPWV (m/s)	9.9 (1.7)	10.4 (1.6)	10.5 (1.6)	0.0002	–	–	–	–
DC (10^−3^ kPa)	13.1 (4.1)	12.1 (4.1)	11.0 (3.6)	< 0.0001	–	–	–	–
ABPI < 0.9	65 (20%)	142 (25%)	40 (32%)	0.007	–	–	–	–
ABPI > 1.4	10 (3%)	10 (2%)	2 (2%)	0.20	–	–	–	–

Values are mean (SD) for normally-distributed continuous variables, n (%) for categorical variables, and geometric mean (interquartile range) for non-normally distributed continuous variables

*“Trend” denotes *p* value for association with frailty status modelled as an ordinal variable

†Statistical comparisons made with variable natural log-transformed

*BMI* body mass index, *eGFR* estimated glomerular filtration rate, *CRP* C-reactive protein, *IL-6* interleukin-6, *tPA* tissue plasminogen activator, *vWF* von Willebrand factor, *hs-cTnT* high-sensitivity cardiac troponin T, *NT-proBNP* N-terminal B-type natriuretic peptide, *CIMT* carotid intima media thickness, *cfPWV* carotid-femoral pulse wave velocity, *DC* carotid distensibility coefficient, *ABPI* ankle brachial pressure index

CRP, IL-6, tPA, D-dimer, vWF, hs-cTnT, NT-proBNP, CIMT, cfPWV and the presence of ABPI < 0.9 were all positively and significantly associated with the presence of frailty in the CVD-free group; DC was negatively and significantly associated with frailty in this group. ABPI > 1.4 showed no significant association with frailty.

In men with prevalent CVD, circulating concentrations of CRP, IL-6, D-dimer, hs-cTnT and NT-proBNP were all positively and significantly associated with frailty. vWF showed a non-significant positive association, whereas tPA showed no clear association.

### Multivariate analysis

Tables 2 and 3 show the multivariate associations between the biomarkers and non-invasive markers with frailty status in men without CVD (Table 2) and in men with prevalent CVD (Table 3). To facilitate comparisons between different variables, odds ratios are given both per unit change in each variable, and per standard deviation increase in each variable (i.e. per 1 z-score increase).

**Table 2:** Associations between frailty status and inflammatory/coagulation/endothelial/cardiac biomarkers, and subclinical CVD in men without clinically-apparent CVD

	Model 1†						Model 2 (*n = 967)*					
	Odds ratio for prefrailty vs robustness (95% CI)	Odds ratio per standard deviation increase (95% confidence interval)	*p*	Odds ratio for frailty vs robustness (95% CI)	Odds ratio per standard deviation increase (95% confidence interval)	*p*	Odds ratio for prefrailty vs robustness (95% CI)	Odds ratio per standard deviation increase (95% confidence interval)	*p*	Odds ratio for frailty vs robustness (95% CI)	Odds ratio per standard deviation increase (95% confidence interval)	*p*
**Inflammatory biomarkers**												
log CRP	1.22 (1.07–1.39)	1.26 (1.08–1.46)	0.003	1.37 (1.14–1.66)	1.47 (1.18–1.82)	0.001	1.05 (0.91–1.22)	1.06 (0.89–1.27)	0.49	1.13 (0.90–1.42)	1.15 (0.88–1.51)	0.30
log IL-6	1.68 (1.35–2.09)	1.46 (1.24–1.70)	< 0.0001	2.26 (1.66–3.09)	1.81 (1.46–2.25)	< 0.0001	1.52 (1.18–1.95)	1.35 (1.13–1.63)	0.001	2.02 (1.38–2.95)	1.67 (1.26–2.20)	0.0003
**Coagulation biomarkers**												
log tPA	1.36 (0.96–1.91)	1.14 (0.98–1.33)	0.08	2.03 (1.22–3.36)	1.36 (1.09–1.70)	0.006	1.32 (0.91–1.91)	1.13 (0.96–1.33)	0.15	1.68 (0.94–3.02)	1.26 (0.97–1.62)	0.08
log D-dimer	1.24 (0.96–1.60)	1.14 (0.98–1.33)	0.09	1.23 (0.85–1.78)	1.13 (0.91–1.42)	0.27	–					
**Endothelial biomarkers**												
log vWF	1.44 (1.05–1.96)	1.19 (1.03–1.37)	0.02	1.73 (1.09–2.75)	1.33 (1.07–1.64)	0.02	1.27 (0.91–1.77)	1.12 (0.96–1.31)	0.15	1.40 (0.83–2.38)	1.18 (0.91–1.51)	0.21
**Cardiac biomarkers**												
log hs-cTnT	1.33 (1.05–1.68)	1.22 (1.04–1.43)	0.02	2.25 (1.54–3.29)	1.76 (1.35–2.29)	< 0.0001	1.13 (0.90–1.42)	1.13 (0.95–1.35)	0.18	1.95 (1.24–3.05)	1.59 (1.16–2.18)	0.004
log NT-proBNP	1.13 (1.01–1.27)	1.17 (1.01–1.36)	0.03	1.26 (1.06–1.15)	1.36 (1.08–1.71)	0.008	1.08 (0.95–1.22)	1.10 (0.93–1.30)	0.27	1.14 (0.94–1.40)	1.19 (0.92–1.54)	0.19
**Imaging markers**												
CIMT	1.12 (0.44–2.88)	1.02 (0.88–1.18)	0.82	3.10 (0.78–12.34)	1.19 (0.96–1.48)	0.11	–					
cfPWV	1.16 (1.05–1.27)	1.27 (1.09–1.49)	0.003	1.11 (0.96–1.29)	1.19 (0.93–1.53)	0.16	1.12 (1.02–1.24)	1.21 (1.03–1.44)	0.02	1.04 (0.88–1.23)	1.07 (0.81–1.40)	0.64
DC	0.96 (0.92–0.99)	0.83 (0.71–0.96)	0.01	0.92 (0.86–0.98)	0.70 (0.54–0.90)	0.006	0.98 (0.94–1.02)	0.90 (0.76–1.07)	0.24	0.92 (0.86–0.99)	0.71 (0.53–0.95)	0.02
ABPI < 0.9 (vs ABPI 0.9–1.4)	1.33 (0.93–1.90)	–	0.12	1.70 (1.00–2.89)	–	0.05	–					
ABPI > 1.4 (vs ABPI 0.9–1.4)	0.61 (0.24–1.55)	–	0.30	0.71 (0.14–3.65)	–	0.68						

Model 1: Adjusted for age, eGFR, smoking status, polypharmacy, cognitive impairment, number of non-cardiovascular comorbidities, social class, BMI class, and systolic blood pressure. Model 2: additionally mutually-adjusted for biomarkers/imaging markers with *p* < 0.05 in Model 1

*BMI* body mass index, *eGFR* estimated glomerular filtration rate, *CRP* C-reactive protein, *IL-6* interleukin-6, *tPA* tissue plasminogen activator, *vWF* von Willebrand factor, *hs-cTnT* high-sensitivity cardiac troponin T, *NT-proBNP* N-terminal B-type natriuretic peptide, *CIMT* carotid intima media thickness, *cfPWV* carotid-femoral pulse wave velocity, *DC* carotid distensibility coefficient, *ABPI* ankle brachial pressure index

†For Model 1, n varies by variable used: log CRP *n* = 1075, log IL-6 *n* = 1085, log tPA *n* = 1074, log D-dimer *n* = 1074, log vWF *n* = 1074, log hs-cTnT *n* = 1075, log NT-proBNP *n* = 1075, CIMT *n* = 1081, cfPWV *n* = 1022, DC *n* = 1076, ABPI *n* = 1032

**Table 3:** Associations between frailty status and inflammatory/coagulation/endothelial/cardiac biomarkers in men with clinically-apparent CVD

	Model 1†						Model 2 (*n = 295)*					
	Odds ratio for pre-frailty vs robustness (95% confidence interval)	Odds ratio per standard deviation increase (95% confidence interval)	*p*	Odds ratio for frailty vs robustness (95% confidence interval)	Odds ratio per standard deviation increase (95% confidence interval)	*p*	Odds ratio for pre-frailty vs robustness (95% confidence interval)	Odds ratio per standard deviation increase (95% confidence interval)	*p*	Odds ratio for frailty vs robustness (95% confidence interval)	Odds ratio per standard deviation increase (95% confidence interval)	*p*
**Inflammatory biomarkers**												
log CRP	1.46 (1.07–1.98)	1.62 (1.09–2.40)	0.02	1.53 (1.07–2.19)	1.74 (1.09–2.78)	0.02	1.59 (1.14–2.22)	1.83 (1.18–2.83)	0.007	1.20 (0.79–1.80)	1.26 (0.74–2.15)	0.39
log IL-6	1.17 (0.69–1.97)	1.13 (0.75–1.70)	0.56	2.01 (1.12–3.63)	1.72 (1.09–2.72)	0.02	0.85 (0.49–1.48)	0.88 (0.58–1.35)	0.57	1.58 (0.83–3.04)	1.43 (0.86–2.37)	0.17
**Coagulation biomarkers**												
log tPA	1.90 (0.84–4.21)	1.34 (0.92–1.96)	0.13	1.39 (0.53–3.66)	1.16 (0.75–1.81)	0.51	–					
log D-dimer	0.67 (0.38–1.18)	0.76 (0.52–1.12)	0.16	1.02 (0.54–1.91)	1.01 (0.66–1.55)	0.96						
**Endothelial biomarkers**												
log vWF	1.19 (0.60–2.35)	1.10 (0.77–1.56)	0.61	1.68 (0.75–3.73)	1.31 (0.86–1.99)	0.20	–					
**Cardiac biomarkers**												
log hs-cTnT	0.80 (0.46–1.38)	0.84 (0.56–1.27)	0.42	4.51 (2.11–9.63)	3.07 (1.74–5.40)	0.0001	0.69 (0.39–1.22)	0.76 (0.49–1.16)	0.20	4.06 (1.84–8.95)	2.84 (1.58–5.12)	0.0005
log NT-proBNP	0.83 (0.64–1.07)	0.73 (0.50–1.11)	0.15	1.13 (0.83–1.54)	1.22 (0.75–1.98)	0.42	–					

Model 1: Adjusted for age, eGFR, smoking status, polypharmacy, cognitive impairment, number of non-cardiovascular comorbidities, social class, BMI class, and systolic blood pressure. Model 2: also mutually-adjusted for biomarkers/imaging markers with *p* < 0.05 in Model 1

*BMI* body mass index, *eGFR* estimated glomerular filtration rate, *CRP* C-reactive protein, *IL-6* interleukin-6, *tPA* tissue plasminogen activator, *vWF* von Willebrand factor, *hs-cTnT* high-sensitivity cardiac troponin T, *NT-proBNP* N-terminal B-type natriuretic peptide

†For Model 1, n varies by variable: log CRP *n* = 297, log IL-6 *n* = 301, log tPA *n* = 294, log D-dimer *n* = 294, log vWF *n* = 294, log hs-cTnT *n* = 298, log NT-proBNP *n* = 298

In men without prevalent CVD, when adjusted for age, BMI class, smoking status, social class, eGFR, and systolic blood pressure (model 1), both CRP and IL-6 showed statistically significant positive associations with pre-frailty and frailty, as did hs-cTnT, NT-proBNP and vWF. Both D-dimer and tPA showed a positive association with pre-frailty and frailty, but this was only statistically significant for tPA. Imaging markers of subclinical atherosclerosis showed differential associations: cfPWV was positively associated with pre-frailty and showed a weaker, non-significant association with frailty; ABPI < 0.9 was associated with greater odds of frailty; DC showed a strong negative association with odds of prefrailty and frailty. ABPI < 0.9 and frailty showed a positive but non-significant association. CIMT showed no association with pre-frailty and a weak, non-statistically significant positive association with frailty. ABPI > 1.4 showed a non-significant association with reduced odds of prefrailty and frailty.

In a model mutually adjusting for log CRP, log IL-6, log tPA, log vWF, log hs-cTnT, log NT-proBNP, cfPWV, and DC, as well as the other covariates in Model 1 (Model 2), only log IL-6, log hs-cTnT and DC remained statistically significantly associated with frailty. cfPWV retained a significant positive association with pre-frailty. The other covariates’ associations with frailty were markedly attenuated.

In men with prevalent CVD, both CRP and IL-6 showed positive associations with frailty, though only CRP retained a significant association with pre-frailty. hs-cTnT, but not NT-proBNP, was positively and significantly associated with frailty. In contrast, log tPA, log D-dimer, and log vWF showed no clear association with either frailty or prefrailty.

In a mutually-adjusted model (log CRP, log IL-6, and log hs-cTnT along with all covariates in Model 1), hs-cTnT remained strongly associated with frailty, whereas CRP retained an association with pre-frailty.

## Discussion

In this cohort of older British men, biomarkers of coagulation, inflammation, endothelial dysfunction, and cardiac strain and damage were strongly associated with prefrailty and frailty in men free of clinical CVD, as were some imaging markers of subclinical vascular dysfunction (carotid distensibility and cfPWV). In contrast, amongst men with CVD, whilst inflammatory biomarkers and troponin were associated with frailty, biomarkers of coagulation and endothelial dysfunction and a biomarker of cardiac injury were not. However, the large confidence intervals in this subgroup suggest that the small number of participants may have limited the statistical power to detect these associations, which cannot be dismissed. In both groups, on mutual adjustment, higher hs-cTnT remained independently associated with higher frailty status (as did higher IL-6 and reduced carotid artery distensibility, in men without overt CVD). Our results suggest that myocardial injury is prominently and independently associated with frailty both in men with, and without, clinically evident cardiovascular disease, and that carotid stiffness is additionally (and independently) associated with frailty status in men without clinically apparent CVD.

### Inflammation and frailty

The strong association between inflammation and frailty in men without CVD seen here is consistent in both direction and strength with those reported in many other cross-sectional studies [6]. Circulating IL-6 levels showed a stronger association with frailty than CRP. IL-6 is an upstream cytokine with broader effects than CRP; these include – in the setting of inflammation – promoting the production of CRP and other acute phase reactants, hepatic lipolysis and muscle proteolysis. IL-6 also regulates other physiological processes, including tissue repair and energy metabolism and may counteract inflammatory cascades in some circumstances [33]. IL-6 levels may therefore reflect a pro-inflammatory state that contributes to frailty, [34] but might also represent adaptive responses to conserve and mobilise energy in a frailty state [35]. The associations between frailty, biomarkers of coagulation, [8] and endothelial dysfunction [9] are likewise consistent with prior reports; however, we then observed that those associations were weakened by mutual adjustment for inflammatory and cardiovascular dysfunction. There is substantial cross-talk between these systems, particularly with inflammation, [36, 37] suggesting that co-activation with, or as a result of, inflammation and/or cardiac injury may explain their association with frailty. In men with CVD, inflammation also showed associations with frailty, though statistical significance was lost on mutual adjustment for both inflammatory biomarkers and hs-cTnT; the small subgroup of men with CVD may have been under-powered to detect a significant association. However, there were no clear associations between coagulation biomarkers, markers of endothelial dysfunction, and frailty in men with CVD.

### Non-invasive CV markers and frailty

Elevated cfPWV, low ABPI, and reduced DC were all associated with greater frailty status. This is generally consistent with prior evidence for PWV and ABPI, [17–19] although we were unable to replicate associations between carotid intima media thickness and frailty [20–22]. To our knowledge, associations between carotid distensibility and physical frailty have not previously been reported. DC was particularly robustly associated with frailty in our study, with a highly statistically significant association persisting despite adjustment for cfPWV and other blood biomarkers associated with frailty – namely, CRP, IL-6, tPA, vWF, hs-cTnT and NT-proBNP. Both DC and cfPWV measure stiffness of arterial walls; the former in the carotid, and the latter mostly in the aorta. The two show differential associations with CVD: carotid stiffness is associated with stroke risk, independently of cfPWV, but not with coronary artery disease (unlike cfPWV) [38]. Carotid stiffness (or stiffness of other elastic arteries, of which the carotid may be a proxy) is hypothesised to lead to increased pulsatile pressure in the cerebral microcirculation, leading to cerebral infarction and haemorrhage; it has also been associated with cerebral small vessel disease and cognitive impairment [39]. Whilst our association between low DC and frailty status was observed in men without a clinical diagnosis of stroke, it is possible that frail men with carotid stiffness had ‘subclinical’ infarcts and/or greater burden of small vessel disease, which might explain the association seen here. Baseline brain imaging would have helped determine this.

### Cardiac markers and frailty

Our results strengthen, and extend, the evidence of relationships between cardiovascular disease and frailty. As in the Rugao Longevity and Aging Study, [14] neurohormonal activation (measured by BNP in their study, and NT-proBNP in ours) was associated with physical frailty, although we found a significant association only in men without existing CVD. NT-proBNP is secreted in response to myocyte stretch, neuroendocrine activation, and myocardial hypoxia, [40] but is also influenced by proinflammatory states [41]. In our study, the association between NT-proBNP and frailty was abolished by inflammatory markers and hs-cTnT, which suggests that inflammation and myocyte injury may well explain the previously-reported association with frailty.

hs-cTnT was robustly associated with frailty status in both subgroups of our study, a finding previously reported in older adults with diabetes [16]; we extend this to a wider population of older men, both with and without cardiovascular disease. hs-cTnT is highly specific for myocyte injury, but not the aetiology of it [42]; hs-cTnT levels in older people are increased by the presence of comorbidities, [43] including non-cardiac conditions. The associations between hs-cTnT and frailty in our study were independent of inflammatory biomarkers, subclinical atherosclerosis (in men without CVD), renal dysfunction, cognitive impairment, and comorbidity burden (measured as an unweighted index). Myocardial injury appears to be associated with frailty, even in men without clinical evidence of cardiovascular disease. However, both NT-proBNP and hs-cTnT levels have been associated with subclinical brain injury on imaging, [44] an important unmeasured factor in our study that may be a confounder.

### Frailty and CVD

Frailty and CVD often coexist [3]. Our findings add to the understanding of the shared common pathways between frailty and cardiovascular disease. Atherosclerosis is a process of vascular endothelial damage, plaque formation, plaque rupture and ongoing healing facilitated by chronic inflammation and the clotting system. A recent meta-analysis has shown that frailty predisposes individuals to incident coronary artery disease, HF and CVD mortality [45] even after adjusting for conventional risk factors. Our findings suggest that this may be due to an underlying subclinical atherosclerosis process, potentially driven by frailty related inflammaging [46]. However, some prospective studies have found significant associations between frailty and incident CVD even after taking inflammation [47, 48] and subclinical atherosclerosis [49] into account, suggesting other mechanisms may exist. We additionally found a cross-sectional association between frailty and hs-cTnT, suggesting that that subclinical myocyte injury might also explain the increased CVD risk associated with frailty; future prospective studies should explore this. CVD also seems to be associated with an increased risk of developing the frailty phenotype [4, 5]. The findings that inflammation and hs-cTnT are associated with frailty in those with overt CVD may help explain why patients with CVD are more prone to frailty.

### Strengths and limitations

This is a relatively large study with multiple detailed cardiovascular and frailty assessments. We were able to simultaneously analyse the effect of multiple different biomarkers and imaging markers, including NT-proBNP, hs-cTnT, and carotid stiffness, for which there is limited extant evidence of their relationship with frailty. We adjusted for important potential confounders, including cognitive impairment, polypharmacy, and multimorbidity. However, this is a cross-sectional study, and causality cannot be directly inferred; nor can the direction of effect be determined, if there is indeed a causal link. Unmeasured confounders may explain some of these associations, including subclinical stroke and cerebral small vessel disease, which may have been undiagnosed but apparent on imaging, had it been available. Our measurement of comorbidity burden was largely based on self-report of diagnoses, which may lack validity, and was not weighted by severity of each illness. In men with CVD, we were unable to account for the severity of their cardiovascular comorbidities; associations between the biomarkers and frailty may simply have been explained by underlying disease severity and secondary frailty. All participants in this study were male and of White British origin; our results may not be generalisable to other groups.

### Implications for future study

Longitudinal studies should investigate the prospective associations between cardiac troponins, subclinical cardiovascular disease, and incident frailty, which, if present, would support the argument that these are causally related. Prospective studies could also account for baseline biomarkers of cardiac injury when examining associations between frailty and CVD risk. Whilst carotid stiffness has been investigated in some detail in relation to dementia and cognitive impairment (‘cognitive frailty’), our novel finding of a robust association with physical frailty should be replicated in other studies. People with frailty but without overt clinical cardiovascular disease are more likely to have subclinical vascular disease or cardiac dysfunction, which is likely to increase their risk of subsequent overt CVD. Vascular assessment may be helpful in this group and could, potentially, help to identify those for whom intervention to reduce cardiovascular risk may be most beneficial.

## Conclusions

In older men without CVD, subclinical atherosclerosis as measured by carotid arterial stiffness, hs-cTnT (a marker of myocyte injury) and IL-6 (a marker of inflammation) were most strongly associated with frailty. In men with CVD, inflammation and myocardial injury, but not coagulation or endothelial dysfunction, were associated with frailty. Our findings suggests that these common pathways might link frailty and CVD.

## Data Availability

Data are available on reasonable request to Lucy Lennon at l.lennon@ucl.ac.uk. The BRHS data sharing policy can be found here: https://www.ucl.ac.uk/epidemiology-health-care/research/primary-care-and-population-health/research/ageing/british-regional-heart-study-brhs-6.

## Abbreviations

ABPI: Ankle-brachial pressure index
cfPWV: carotid-femoral pulse wave velocity
CIMT: Carotid intima-media thickness
CRP: C-reactive protein
CVD: Cardiovascular disease
DC: Carotid distensibility coefficient
hs-cTnT: high-sensitivity cardiac troponin T
IL-6: Interleukin-6
NT-proBNP: N-terminal pro B-type natriuretic peptide
tPA: tissue plasminogen activator
vWF: von Willebrand factor
